# Factors influencing the migration of Iranian healthcare professionals: A qualitative study

**DOI:** 10.1371/journal.pone.0199613

**Published:** 2018-06-27

**Authors:** Heshmatollah Asadi, Batoul Ahmadi, Saharnaz Nejat, Ali Akbari Sari, Ali Garavand, Abdollah Almasian Kia, Mojtaba Hasoumi

**Affiliations:** 1 Department of Health Management and Economics, School of Public Health, Tehran University of Medical Sciences, Tehran, Iran; 2 Department of Public Health, School of Public Health, Lorestan University of Medical Sciences, Khorramabad, Iran; 3 Department of Epidemiology and Biostatistics, School of Public Health, Tehran University of Medical Sciences, Tehran, Iran; 4 Department of Healthcare Management, School of Health Management and Information Sciences, Iran University of Medical Sciences, Tehran, Iran; 5 Department of Healthcare Economics, School of Management and Information Sciences, Shiraz University of Medical Sciences, Shiraz, Iran; 6 Health Management and Economics Research Center, Iran University of Medical Sciences, Tehran, Iran; University of the Witwatersrand, SOUTH AFRICA

## Abstract

**Background:**

The migration of healthcare specialists from developing countries has increased in recent years. This has caused a rapid reduction in the access to and quality of healthcare services in such countries. The aim of this study is to evaluate the factors affecting the migration of specialist human resources in Iran’s healthcare system.

**Methods:**

This is a qualitative study, which was carried out through semi-structured interviews between 2015 and 2016. For sampling, purposive sampling method with maximum variation sampling was used. Further, data saturation was observed by conducting 21 interviews, and data analysis was performed using the MAXQDA10 content analysis software.

**Results:**

Factors affecting the migration of specialists were classified into five key themes, including structural, occupational, personal, socio-political and economic factors. These themes consisted of 12 categories and 50 subcategories. The most important factors affecting the migration of our study population were structural issues, occupational problems, and personal concerns.

**Conclusion:**

Identification of factors influencing migration is the first step to prevent the migration of specialist human resources. Implementing the recommendations proposed in this study would assist to prevent migration of medical professionals.

## Introduction

Human resources (HR) are essential for the evolutions of all nations; and a depletion of these resources delays the process of growth and development [1]. Human resources are also the most important inputs of the healthcare system [2] and play a vital role in providing healthcare services. Thus, clear and appropriate human resources policies are needed in order to advocate countries in realizing health equity as an objective which subsequently contributes to the universal call of the”Sustainable Development Goals (SDGs) [3].

However, the importance of human resources has been neglected in the healthcare sector of developing countries [4]. The migration of health specialists has risen steadily in recent years [5]. It is noteworthy that the World Health Organization stated in 2006 that the worldwide shortage of doctors, nurses, midwives, and other health professionals is by approx. 4.3 million workers [6].

This has resulted in a number of negative consequences. First, it entails the rapid reduction of access to and the quality of healthcare services which is most pronounced in rural areas and public sector [7]. Such migration has led to reduced number of health personnel, disruption of services, greater dissatisfaction, allowing other staff to resign, increased waiting time for patients seeking care, rising costs of healthcare, loss of experienced teachers, loss of active population, as well as increase of dependency [8–10].

A comprehensive literature review carried out by the research team has revealed that in Iran, unfortunately, there is no comprehensive and documented information neither on the extent nor the number of immigrants of different major and fields, with health care workforce being no exception. In some cases, there is also no evidence regarding the number of graduated and ready to work individuals. Lack of information and detailed statistics in these areas are quite evident. The net migration rate of Iranian people during 2000–2010 was reported about 1.493 million people, indicating the greater outflow of the country's population to other countries compared to immigrants entering into Iran from other countries [11]. In 2009, Iran was ranked first in terms of migration rate of skilled and educated people amongst 91 developed and developing countries [12].

Allaedini et al. conducted a study to determine the extent and reasons of the inclination to migrate among Iranian physicians. This study was performed on a sample of 5482 physicians. The results showed that the average rate of physicians who are willing to immigrate was 53.3% [13]. Also, the findings of another study carried out on the students of Iran University of Medical Sciences categorized the factors affecting migration of students as follows: economic, educational, administrative, professional, social and cultural factors, besides the phenomenon of globalization [14].

Haghdoust et al, conducted a study to investigate the documents related to scholarships granted to Iranian students by the Iran's Ministry of Health and Medical Education. They found that the rate of those graduated students who have not returned to the country was around 40%. Results of this study indicated that the lowest and the highest rate of students returning to the country was related to those who completed their study in North American (10%) and European (70.2%) countries, respectively [15]. In another study, Sohbatiha et al, conducted a study whose research community included Iranian academics with a position in the scientific board of the 10 top American universities. In this study, Scopus was used to collect required information. Results showed that of all professors with a role as faculty member 141 professors were Iranian, most of whom (about 40%) were active in the field of medical sciences [16]. In Iran, limited studies have focused on defining factors affecting the migration of health care human resources. The aim of this study is to determine the factors influencing the migration of specialist human resources in the health system of Iran.

## Materials & methods

This is a qualitative study, carried out between December 2015 and September 2016. The inclusion criteria were as follows:

1) Actual migration of individuals or their desire to emigrate; 2) being Iranian; 3) having a history of living in Iran before emigrating; and 4) education in graduate levels of science fields or at least five years of work experience in the field of medical sciences. Purposeful sampling with maximum variation sampling (in terms of field of study) was used to select the participants in the study. Two individuals were excluded due to personal reasons.

Different methods were used to access the participants. In one, we used the university website to contact faculty members of different scientific boards of three large universities, including Tehran, Iran, and Shahid Beheshti universities Via academic email. They were asked to introduce those people who meet the inclusion criteria of the present study and whether they wanted to participate in the study. Another way to find participants for the study was through personal contacts to those who had referred to the human resources department of Iranian Ministry of Health for translating their educational degree. We also asked the participants to introduce other eligible people who were willing to participate in the study.

Data collection was carried out through semi-structured interviews. This study was reviewed and approved by the Ethic Committee of Tehran University of Medical Sciences, Iran (No. IR.TUMS.REC.1394.131) before the study began. The objectives of the research and characteristics of the research team were explained to interviewees beforehand. All participants were informed about the purpose, type of study, ways of publishing data and the voluntary nature of participation. Data were collected only from those who voluntarily participated in this study. For those in Iran, interviews were carried out face to face during recess time. Also, Skype was used to conduct interview for those Iranian living abroad. The interviews were conducted by two members of the research team (H.A., B.A.) so that each single interview was conducted by only one researcher. An interview guide was prepared using the opinions of experts and the research team based on the objective of study (S1 Appendix). Each interview session lasted for between 30–120 minutes. Statements of individuals were recorded using Sony icd-px33 voice recorder in addition to taking notes.

The answers were reviewed immediately after the end of each interview and were then typed verbatim so that saturation point could be achieved through the level of similarity of individual responses. In this study, data saturation was met after 21 interviews; however, in order to make sure of data saturation, the research team agreed to perform three additional interviews.

Data analysis was done using MAXQDA10 software, and Conventional Content Analysis approach was used for data analysis [17]. Content analysis is a suitable method to achieve valid and reliable results from text data [18]. Transcripts of the interviews were initially read carefully to analyse the data. After obtaining an overview of the interviews, each transcript was re-read word by word in order to extract codes from the interviews. The final analysis was done after determination of codes, and the relation between code and categories was then established. The agreement on codes was concluded and interviews were reviewed by the research team (Peer check). Member checking was also performed to ensure accuracy of data collected.

## Results

A total of 24 interviews with eight females and 16 males were carried out in this study. The participants” characteristics are presented in Table 1.

**Table 1:** Characteristics of the participants in the study.

Characteristics		Number
Degree of education	MD, PhD, doctoral student	14
	MS, MA	6
	BS, BA	4
Fields of study	Nursing	5
	Physiotherapy	5
	Medicine	3
	Health Management	2
	Health Informatics	2
	Physiology, nutrition, health economics, public health, environmental health, professional health, clinical psychology	One of each
Country	Iran	13
	Australia	4
	USA	3
	Belgium, Canada, Italy, The Netherlands	One of each
TOTAL		24

Factors affecting the migration of specialists were classified into five themes—structural, occupational, personal, socio-political and economic—after carrying out content analysis of interviews. These five themes were presented in form of 12 categories and 50 subcategories (Tables 2 and 3).

**Table 2:** Factors affecting the migration of human resources in the health system of Iran–Themes 1–3.

Theme	Category	Subcategory
**1: Structural**	1: Problems arising from Ministry of Health (37.5%)^a^	Wrong policies of the Iranian Ministry of Health
		Restrictive regulations
		Lack of accountability of the Ministry of Health
		Lack of permanent and appropriate supervision of the Ministry of Health
	2: Lack of optimal use of the knowledge and experiences of graduates (45.83%)	Unfavourable conditions for the development of the abilities of individuals
		Feeling of not being useful to society
		Poor management of graduates
	3: Educational system problems (45.83%)	Unsuitable educational environment
		Comparing academics in Iran and that in other countries
		Problems of students acceptance system
		Poor management in the education system
	4: Problems in the field of research (20.83%)	Disregard or lack of application of results from health-related research
		Insufficient financial resource allocation
		Poor management of financial resources in research
		Restrictions on research process
**2: Occupational**	5: Workplace problems (70.83%)	Dissatisfaction with the behaviour of higher officials
		Psychological pressures at the workplace
		Low welfare facilities
		Dissatisfaction with salary
		Problems of job security
		Uncertainty of the job description
		Inappropriate occupational position and weak employment protection
		Inappropriate recruitment processes and selection (e.g. selection criteria)
	6: Discrimination between fields (37.5%)	Uneven distribution of employment opportunities among different fields
		Insufficient attention to some fields
		Physician-controlled tendency
		Disparity in job security in different fields
		Unfair income gap between different fields
	7: Strengthening the scientific status (45.83%)	More professional experience
		Strengthening level of foreign language skills (Primarily English knowledge)
**3: Personal**	8: Gaining new experiences (37.5%)	The experience of studying in foreign universities
		The experience of living in another country
	9: Achieving a better life (54.16%)	Hope to make further progress abroad
		Better equipment and facilities abroad
		Dissatisfaction with the quality of life in Iran
	10: For children (16.66%)	The future of children
		Children’s safety

^a^ Numbers in parentheses indicate the percentage of the participants who mentioned one of the theme factors as their reason to migrate.

**Table 3:** Factors affecting the migration of human resources in the health system of Iran–Themes 4–5.

**4: Socio-political**	11: Socio-political limitations (87.5%)	Restrictions on individual and social freedoms
		Social security status
		Ideological and religious issues
		Lack of meritocracy
		Cultural status
		Unbalanced development in different regions of the country
		Political situation
		Creating a dual personality
		Cultural restrictions for women
**5: Economic**	12: Economic & financial problems (70.83%)	Economic situation of the country
		Inadequate income
		The income gap with other countries
		Less income compared to other professions inside the country

^a^ Numbers in parentheses indicate the percentage of the participants who mentioned one of the theme factors as their reason to migrate.

### A) Structural factors

Structural factors are difficulties and limitations in the structure of health system organizations and these problems are due to faulty structure of such organizations or incorrect decisions of Ministry of Health or its”organizational subsidiaries. Structural factors consist of four categories, including problems caused by the Ministry of Health, lack of using knowledge and experience of graduates, improper functioning of the educational system, and problems in the field of research.

Some of the individuals (37.5%) pinpointed that difficulties in the area of regulations and policies arising from the Ministry of Health are one of the reasons to migrate. One opined:

“…*restrictive regulations of the Ministry of Health did not let us do our work* … *you must go through many doors to get the approval of teaching an academic field first*, *the university*, *then to the ministry*, *then to the Cultural Revolution Council*, *and again to the university*, *which is finally rejected in majority of cases and this would take several years*” (P. 20). “*I had a clinic in Tehran*, *and I was so annoyed when I wanted to take permission for my medical office practices and my wife always told me we will leave one day and will then live in comfort*” (P. 11).

Some of the participants in the study (45.83%) believed that there is no proper utilization of knowledge and abilities of people inside the country, for example:

*“everyone ignores you after so many years of studying and no one cares about you*” (P. 15). “… *It hurts people when you force them to go to a deprived city or province and you do not provide proper services for them”* (P. 13).

Problems related to the health training system have been also mentioned by some interviewees (45.83%) as a factor influencing the decision to migrate. The education environment in the country is inappropriate, according to some of the participants. Also, some of the participants believed that accepting students is not based on a specific criterion, and there is an oversupply of students in some study fields. One of the individuals states:

*“Their method of managing educated people is wrong and they will receive no feedback of their efforts*, *which means they train individuals but do not use them”* (P. 13).

Another reason for the migration of people, especially those in graduate levels, was problems in the field of research. This factor has been pinpointed by 20.83% of the interviewees: “*You want to do your research but you are caught off guard by monetary and budget problems”* (P. 11). “…Decisions are taken out of our control regardless of to our research outputs… the majority have come to the conclusion that their efforts have been fruitless” (P. 1).

### B) Occupational factors

Occupational factors are factors attributable to the job and the education field of an individual. They include categories of problems related to work environment, perceive discrimination across academic fields, and strengthening scientific status. One of the factors referred to by 70.83% of the interviewees was problems related to the work environment. Some quotes are mentioned below:

“*There are no circumstances for well-being in the workplace*, *there is no conditions for recreation in the hospital environment*” (P. 14). “*I am not officially hired and I do not have job security and I have to work at private hospitals where they will fire you at the slightest dissatisfaction*” (P. 9). “*My father is not the financier and shareholder of a hospital*, *my father is not a university professor*, *so I will not have job security*. *My job security can be a place where I have connections and I know such people*” (P. 18). “*This fact has become routine in Iran that once a new boss comes to power*, *other professionals of lower ranks are changed*, *too …… and it did not make my job stable and each official wants to change your position according to their preferences*” (P. 22).

Some of the participants in the study (35.5%) perceived the discrimination across academic fields as an important factor. This matter is more pronounced in relation to the greater attention given to doctors, for example:

“*I believe there is no opportunity for progress in other fields and even the method of treatment is different; and only medical staff*, *especially doctors*, *are important for the health system*” (P. 7). Issues of income disparity and promotions were also among factors that were stated by interviewees: “*Doctors are given more chance in policy makings and enjoy benefits in positions as well as income*” (P. 1). Other participants stated: “*Only doctors have managerial positions in our country*” (P. 4). “*My main reason for immigration is the fact that health is worthless … This is really disturbing*” (P. 3).

Some of the interviewees (45.83%) stated that knowledge promotion was the main driver in their decision to leave their country. One of the participants in the study who lived abroad stated that:

“*My reasons were my great passion for further studies mainly out of the country because such a condition was not prepared in my home country*” (P. 11).

### C) Personal factors

Factors that are the results of individual opinion and are due to tendencies arising from experiences or desires of individuals or their families. Personal factors included categories of experience and achieving better opportunities for themselves and their children.

The inner tendency to experience studying and living abroad was also among factors affecting migration which was referred to by some participants (37.5%) in this study. One of the interviewees who lived abroad stated that:

*“I always wanted to live here … so regardless of good or bad situations in Iran*, *I wanted to have such an experience in my life*” (P. 21). “*Personally*, *I wanted to continue studying abroad*” (P. 17).

About half of the participants (54.16%) considered the desire to achieve a better life as the reason for their migration or the tendency to migrate:

“*Another reason is the condition I am living in*. *It is not what I want*, *and perhaps it does not match my thoughts or ideas*” (P. 14). “*Based on what I know about myself*, *I think I can get what I want if I go to another country*” (P. 7).

On the other hand, some of the interviewees (16.66%) considered the future of their children as a key driver for their migration *“I do not want to raise the pressure on my children and their insecurity*” (P. 10). This factor was considered to be important by those who were older. “… *I wish my children to grow up in a society where they can develop and show their abilities*” (P. 6).

### D) Socio-political factors

Factors related to the political situation in the country or social factors (cultural, religious, social security, etc.). Socio-political factors include one category and 9 subcategories.

This factor is one of reasons of migration referred to by 87.5% of the participants in the study. One of the individuals living outside Iran described social security as:

“*For example*, *if my child goes out to play*, *I am relieved that no danger threatens him… there will even be no problem if the door is left open…*. *This shows the security*” (P. 16).

### E) Economic factors

Factors caused by economic problems of the country or inadequate financial incomes of individuals inside the country. Economic factors included the categories of economic and financial reasons.

Financial and economic problems were referred to as the cause of migration and tendency to emigrate by the majority of individuals (70.83%). Inadequate income as well as lesser salaries relative to other economic sectors were among factors affecting emigration of individuals considered in the research. Some of the participants in the study considered their income to be unfair in proportion to the level of their education and the efforts they put forth at work compared to other professionals in the country. The income inequality was also considered to be a factor influencing emigration of staff in fields of medical science. Also, the income difference between medical staff inside the country and the destination country has been stated as one of the drivers for emigration.

“*One of my main reasons for immigration was difference between income of nurses in our country and that of those in Canada*, *America*, *and other countries*, *and this difference is evident and immigration is worth it*” (P. 4).

## Discussion

This qualitative study aimed to determine the factors affecting the migration of specialist human resources in the health system of Iran in 2015. The findings of this study showed that the most important reasons for the migration had their root in structural, occupational, personal, socio-political and economic factors. Mainly, the structural issues, occupational problems and personal concerns were pointed out by most individuals as the top drivers for migration.

Iran suffers a shortage of manpower in the healthcare sector [6]. In addition, Iran is among the countries with high yearly rates of migration [12]. A total of 39.6% of medical science students who have been granted scholarship abroad have not returned to the country [15] to the best of our knowledge, no qualitative study has been conducted yet on the factors influencing migration in the healthcare sector in Iran. Only one study has been carried out on the causes of migration among healthcare professionals during the last 10 years. This study was conducted on students who were living in Iran [14], but there has been no study on individuals who have left the country.

Based on the results of the present study, the structural problems of the health system (including problems arising from the Ministry of Health, lack of optimal use of knowledge and experiences of graduates, problems with the education system, and problems in the field of research) are the most important factors influencing the migration of health specialists in Iran. The failure to appreciate the efforts made by health care staff is a leading cause to leave the organization [19]. Low educational quality in the country of origin, pursuing higher education quality in the countries of destination [20], and seeking better research budgets and funding were also reported in the literature as the main factors influencing migration s [21]. Results of these studies are consistent with those of the present study. In this study, structural problems were the most important influencing factor in migration.

Occupational factors affect the migration of specialists, too. This factor includes three subcategories (strengthening scientific status, discrimination across academic fields, and work environment problems). Another study carried out in Iran concluded that promotion of academic and professional growth, lack of job opportunities inside the country, and better job opportunities in other countries were among factors affecting migration among the faculty members of Tehran University [22], which was inconsistent with the findings of the present study. Another factor in the study, which was highly emphasized by non-medical specialists, was paying more attention to doctors and discrimination across academic fields. This factor has not been marked as influential in studies carried out in other countries which indicates the difference between Iran and other countries. It is recommended to conduct further studies in this area.

Yet, another set of key factors influencing the migration of specialists was attributable to personal factors, which include achieving a better life for themselves and their children. Results of the study of Nimah Humphries also showed that personal factors are one of the reasons of migration [21]. Also, Posy Bidwell showed in his study that one of the reasons for migration to Great Britain is the experience of working in the healthcare system of a First World country and personal development factors [23].

Socio-political factors are one of the other important factors affecting the migration of healthcare specialists in Iran. Various literature have also pinpointed that political environment and socio-political problems accelerate the migration of healthcare workers [10, 24]. Doctors who migrate from Pakistan also believe that there is no room for the promotion of individuals based on their abilities and merits in their country of origin [25].

Socio-political factors entail economic factors which are marked among the most important factors affecting the brain drain in the study of Nouri Hekmat et al. [14]. The inconsistent results reported by this study and that of ours could be due to the fact that our study has been conducted on people who have emigrated or want to emigrate but the study of Nouri Hekmat et al. was carried out on students. It was also determined in the study of Allaeddini et al. that inadequate income, high cost, high inflation, and economic instability are among the most important factors affecting the willingness of Iranian physicians to migrate [13].

## Limitations of the study

One of the limitations in this study was accessing to experts who had emigrated from Iran and were living in other countries which made face-to-face interviews impossible. Also, as the budget of this research was limited, interviews with people outside Iran were done on Skype. The time differences between Iran and other countries was another source of limitation in this study. Thus, some interviews had to be conducted in the early hours of Iran time. In some cases, participants in the study who were living in Iran were not willing to easily provide information to the researchers so that much of the interview time was devoted to fix this problem. Furthermore, the number of interviews was increased in some cases. So deserves attention that caution should be taken when interpreting results related to socio economic factors and its generalization as this should not be done without considering the above mentioned limitations.

## Conclusions

The findings of this study could provide policymakers, health system managers, and higher-level policymakers with awareness on factors affecting human resources migration and reveal strategic reasons influencing the migration of human resources. Given low rate of return after migrants, state authorities should focus on preventing further migration of human resources. In this regard, we recommend authorities to use the following three programmes to reduce the rate of emigration:

### 1. Short-term programme in relation to occupational factors

We recommend to consider actions such as attention to all health professions, qualified appointment setting for healthcare organizations, and reviewing welfare programs.

### 2. Medium-term programme in relation to structural and economic factors

We recommend actions such as equitable distribution of income across different professions in the healthcare system, curricula revision of workforce, and providing sufficient research budget, to be considered in relation to these factors.

### 3. Long-term programme in relation to personal and social factors

Providing more attention to personal and social freedoms, facilitating ties with other countries and universities, placing practical emphasis on meritocracy, and setting qualification criteria for managing positions, and informing the method of selecting managers for these occupations are among factors which must be considered in this regard.

Further, a complementary study in the field of prevention strategies of migration of specialists in the healthcare system of Iran could provide a suitable route for future research on this topic.

## Supporting information

S1 Appendix Interview guide in Persian and English languages. (DOCX)
