# Supplementary material for: Factors influencing the migration of Iranian healthcare professionals: A qualitative study
Source: PLoS One. 2018 Jun 27;13(6):e0199613. doi: 10.1371/journal.pone.0199613 (PMC6021092; doi:10.1371/journal.pone.0199613)
Supplement: S1 Appendix — (DOCX) [file pone.0199613.s001.docx]

**Interview guide in Persian and English languages**

- Survey questions used in the study in the original language (Persian)

به نام خدا

1-رشته ی تحصیلی ­تان چیست؟ لطفا آخرین مقطعی تحصیلی­تان را نیز بیان نماید؟

2-اکنون در کدام کشور ساکن هستید (این سوال از ساکنین خارج از کشور پرسیده می­شد)؟

3- در صورت تمایل اگر اطلاعاتی در رابطه با خودتان هست که فکر می­کند برای مطالعه ما مفید باشد لطفا آن را نیز بیان نماید؟

3-دلایل مهاجرت (تمایل به مهاجرت) شما چیست (چه بود)؟ لطفا به صورت باز توضیح دهید.

4- کدام نیازهای شما اگر در داخل کشور تامین شود احتمال مهاجرت شما کمتر می­شود؟ در صورت امکان به صورت باز توضیح دهید.

- Survey questions used in the study in English language

In the name of God

1. What is your major / career? Please specify your educational grade.
2. What country are you currently living in? (This question was to be asked from those residents out of Iran)
3. Is there any information about yourself that is thought to be useful to our study? Answering this question is OPTIONAL.
4. What has / have been (are) your reasons for immigration (wanting to immigrate)? Please answer this question openly and completely as possible.
5. Which of your needs should be (have been) met in Iran to make (that could have made) it less likely for you to immigrate? Please answer this question openly and completely as possible.
